# Sister Mary Joseph's nodule at a University teaching hospital in northwestern Tanzania: a retrospective review of 34 cases

**DOI:** 10.1186/1477-7819-11-151

**Published:** 2013-07-05

**Authors:** Phillipo L Chalya, Joseph B Mabula, Peter F Rambau, Mabula D Mchembe

**Affiliations:** 1 Department of Surgery, Catholic University of Health and Allied Sciences-Bugando, Mwanza, Tanzania; 2 Department of Pathology, Catholic University of Health and Allied Sciences-Bugando, Mwanza, Tanzania; 3 Department of Surgery, Muhimbili University of Health and Allied Sciences, Dar Es Salaam, Tanzania

**Keywords:** Sister Mary Joseph’s nodule, Clinicopathological, Pattern, Treatment outcome, Tanzania

## Abstract

**Background:**

Sister Mary Joseph’s nodule is a metastatic tumor deposit in the umbilicus and often represents advanced intra-abdominal malignancy with dismal prognosis. There is a paucity of published data on this subject in our setting. This study was conducted to describe the clinicopathological presentation and treatment outcome of this condition in our environment and highlight challenges associated with the care of these patients, and to proffer solutions for improved outcome.

**Methods:**

This was a retrospective study of histologically confirmed cases of Sister Mary Joseph’s nodule seen at Bugando Medical Centre between March 2003 and February 2013. Data collected were analyzed using descriptive statistics.

**Results:**

A total of 34 patients were enrolled in the study. Males outnumbered females by a ratio of 1.4:1. The vast majority of patients (70.6%) presented with large umbilical nodule > 2 cm in size. The stomach (41.1%) was the most common location of the primary tumor. Adenocarcinoma (88.2%) was the most frequent histopathological type. Most of the primary tumors (52.9%) were poorly differentiated. As the disease was advanced and metastatic in all patients, only palliative therapy was offered. Out of 34 patients, 11 patients died in the hospital giving a mortality rate of 32.4%. Patients were followed up for 24 months. At the end of the follow-up period, 14(60.9%) patients were lost to follow-up and the remaining 9 (39.1%) patients died. Patients survived for a median period of 28 weeks (range, 2 to 64 weeks). The nodule recurred in 6 (26.1%) patients after complete excision.

**Conclusion:**

Sister Mary Joseph’s nodule of the umbilicus is not rare in our environment and often represents manifestation of a variety of advanced intra-abdominal malignancies. The majority of the patients present at a late stage and many with distant metastases. The patient's survival is very short leading to a poor outcome. Early detection of primary cancer at an early stage may improve the prognosis.

## Background

Sister Mary Joseph’s nodule is a metastatic tumor deposit in the umbilicus and often represents advanced intra-abdominal malignancy with dismal prognosis [1]. The term “Sister Mary Joseph’s nodule” was coined by Sir Hamilton Bailey in his book, *Physical Signs in Clinical Surgery*, in honor of Sister Mary Joseph, Dr. William Mayo’s surgical assistant in the early days of the Mayo Clinic. Sister Mary Joseph identified the relationship between umbilical nodules and advanced intra-abdominal malignancy [2-4].

Sister Mary Joseph’s nodules are usually malignant and the most common primary site is an abdomino-pelvic tumor. It is estimated that 1 to 3% of the abdomino-pelvic malignancies metastasize to the umbilicus [5-7]. In men, the commonest primary site is the gastro-intestinal tract of which the stomach is the single most common entity, whereas gynecological malignancies – particularly epithelial ovarian tumors – are the most common primary sites in women. In 15 to 30% of patients, the source of the primary tumor remains unknown [8-10]. The most common histological type is adenocarcinoma (75%), more rarely squamous cell carcinoma, followed by undifferentiated tumors or carcinoids that can metastasize to the umbilicus [11-14]. Sometimes umbilical nodules may be benign (for example, endometriosis, fibroma, epithelial inclusion cysts, foreign body granuloma, keloid, myxoma), and occasionally it may be a primary malignant tumor of the umbilicus (for example, melanoma, squamous or basal cell carcinoma, sarcoma) [15]. Benign umbilical nodules are called Pseudo Sister Mary Joseph’s nodules [16]. Therefore, a histological or cytological study of the umbilicus is not only mandatory, but it also guides the clinician to search for the potential primary site.

The presence of an umbilical metastasis indicates a poor prognosis and is a sign of advanced malignant disease. The survival of these patients has been reported to range from 2 to 11 months from the time of initial diagnosis [2,6,11,17,18]. Because Sister Mary Joseph’s nodule may sometimes be the first and only sign of an internal neoplasm and prognosis is mostly poor, diagnosis has to be confirmed in the early stages to improve average survival.

The diagnosis of Sister Mary Joseph’s nodule is usually delayed due to non-specific symptoms that are often misinterpreted as benign umbilical nodules. The lack of suspicion and general reluctance to perform invasive investigations contributes to late diagnosis. The delay in diagnosis and, consequently, treatment leads to the extremely poor prognosis associated with this disease. A high index of suspicion is required in the management of Sister Mary Joseph’s nodule and all suspected lesions should be biopsied.

The management of Sister Mary Joseph’s nodule in resource-limited countries like Tanzania poses major therapeutic challenges which need to be addressed. Late presentation with advanced lesions coupled with a lack of therapeutic facilities such as adjuvant therapy services are among the hallmarks of the disease in developing countries. The outcome of patients with Sister Mary Joseph’s nodule has been poor because the majority of these patients present late to the hospital with advanced stage. This is partly due to paucity of local data regarding this condition and lack of community awareness on the importance of early reporting to hospital for early diagnosis and treatment. This study was conducted to describe the clinicopathological pattern and treatment outcome of patients with Sister Mary Joseph’s nodule treated at our center and to highlight challenges associated with the care of these patients and proffer solutions for improved outcome.

## Methods

Between March 2003 and February 2013, a retrospective study of histologically confirmed cases of Sister Mary Joseph’s nodule was conducted at Bugando Medical Center. Bugando Medical Center is a tertiary care and teaching hospital for the Catholic University of Health and Allied Sciences-Bugando (CUHAS-Bugando) in the Lake and Western Zones of the United Republic of Tanzania. It is situated along the shores of Lake Victoria in Mwanza City. It has 1000 beds and serves as a referral center for tertiary specialist care for a catchment population of approximately 13 million people. The hospital has a newly established oncology department which provides care for all patients with histopathologically proven cancers. However, the department does not provide radiotherapy services at the moment due to the lack of this facility at our center. As a result, patients requiring this modality of treatment have to travel long distances to receive radiotherapy at the Tanzania Tumor Center, located a considerable distance from the study area.

The study population included all patients who presented to Bugando Medical Center with histologically confirmed Sister Mary Joseph’s nodule during the study period. Patients with incomplete data were excluded from the study.

The details of patients were retrieved from the patient files kept in the medical record department, the surgical wards, operating theatre and histopathology laboratory. The data collected included age, gender, clinical presentation, size of umbilical nodules, timing of discovery of nodules, investigations carried out to look for the primary cancer, sites of origin of primary cancer, therapeutic options, and duration of survival. The diagnosis of Sister Mary Joseph’s nodule was confirmed in all patients by umbilical biopsy, and a histopathological review showed various malignancies metastasizing to the umbilicus. Searching for the primary cancer was aided by gastrointestinal endoscopies (that is,oesophagogastroduodenoscopy)and imaging modalities (that is, abdominal ultrasound, computed tomography (CT) scan and barium studies). Advanced diagnostic investigation such as magnetic resonance imaging (MRI), endoscopic retrograde cholangiopancreatography (ERCP), percutaneous transhepatic cholangiopancreatography (PTC), positron emission tomography (PET) and a panel of tumor markers was not done in any of our patients as these facilities are not available at our center. Patients were followed up in the Surgical Outpatient Department and their death and recurrence of the disease was documented.

### Statistical data analysis

Statistical data analysis was performed using SPSS computer software version 17.0 (SPSS, Inc., Chicago, IL, USA). Data were summarized in the form of proportions and frequency tables for categorical variables. Continuous variables were summarized using median and ranges.

### Ethical consideration

Ethical approval to conduct the study was sought from the CUHAS-Bugando/Bugando Medical Center joint institutional ethic review committee before the commencement of the study

## Results

**Figure 1:**
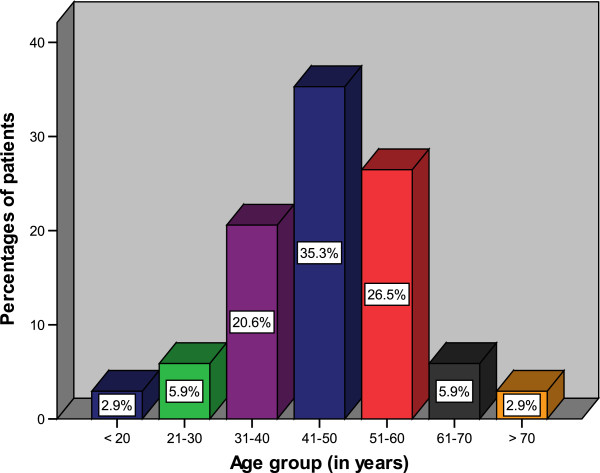
Distribution of patients according to age group.

During the study period, a total of 34 patients were enrolled in the study. There were 20 (58.8%) males and 14 (41.2%) females with a male to female ratio of 1.4:1. The age of patients at presentation ranged from 19 to 73 years with a median age of 46 years. The modal age group was 41–50 years (Figure 1). The vast majority of patients (28, 82.4%) came from the rural areas located a considerable distance from Mwanza City and most of them (30,88.2%) had either primary or no formal education.

The duration of illness ranged from 1 to 6 months with a median of 3 months and the majority of patients (25,73.5%) presented between 2 and 4 months of onset of illness. Table 1 shows the distribution of patients according to clinical presentation. All patients presented with umbilical nodules. The nodules were discovered at the initial clinical examination in 26 (76.5%) patients and in the other 8 (23.5%) patients it was noticed during surgery. The umbilical nodules were tender and ulcerated in 20 (58.8%) patients, hard in all, and the median nodule size at presentation was 3cm (range 2 to 5 cm).The vast majority of patients (70.6%) presented with a large nodule, >2 cm in size. One patient with an umbilical nodule reported an operation for colonic cancer 3 years previously. The stomach was the most common location of the primary tumor, accounting for 41.1% of cases. Adenocarcinoma was the most frequent histopathological type occurring in 88.2% of patients. Most of the primary tumors (52.9%) were poorly differentiated (Table 2).

**Table 1:** Distribution of patients according to clinical presentation

**Clinical presentation**	**Frequency**	**Percentage**
Abdominal pain	30	88.2
Abdominal mass	26	76.5
Weight loss	25	73.5
Constipation	14	41.2
Vomiting	12	35.3
Ascites	10	29.4
Rectal bleeding	4	11.7
Hematemesis/ melena	1	2.9
Other rare symptoms	6	17.6

**Table 2:** Distribution of patients according to nodule size, primary tumor location, histopathological type and tumor grade

**Study variables**	**Frequency**	**Percentage**
**Umbilical nodule size**		
• ≤2 cm	10	29.4
• >2cm	24	70.6
**Primary tumor site**		
• Stomach	14	41.2
• Colorectum	3	8.8
• Pancreas	2	5.9
• Ovary	2	5.9
• Gall bladder	1	2.9
• Anus	1	2.9
• Unknown	11	32.4
**Histopathological type**		
• Adenocarcinoma	30	88.2
• Non-Hodgkin's lymphoma	1	2.9
• Squamous cell carcinoma	1	2.9
• Anaplastic carcinoma	1	2.9
• Cholangiocarcinoma	1	2.9
**Tumor differentiation**		
• Well differentiated	5	14.7
• Moderately differentiated	6	17.6
• Poorly differentiated	18	52.9
• Not documented	5	14.7

Surgical intervention with or without adjuvant therapy was performed in 28 (82.4%) patients. The remaining 6 (17.6%) patients were unfit for surgery and only an incisional biopsy from the umbilical nodule was taken to confirm the diagnosis. Table 3 shows the type of surgical procedure performed. Chemotherapy was given to only 5 (14.7%) patients who had non-Hodgkin lymphoma, colonic and ovarian carcinoma. Only 1 patient (2.9%) who had squamous cell carcinoma was referred to the Ocean Road Cancer Institute for possible radiotherapy. However, due to final constraints this patient could not get access to this form of treatment and they were lost to follow-up.

**Table 3:** Distribution of patients according to the type of surgical procedure performed (N= 28)

**Type of surgical procedure performed**	**Frequency**	**Percentage**
Umbilical nodule excision	28	100
Gastro-jejunostomy	10	35.7
Gastrectomy	5	17.9
Hemicolectomy	3	10.7
Salpingo-oophorectomy	2	7.1
Cholecystectomy	1	3.6

Out of 34 patients, 11 patients died in the hospital giving a mortality rate of 32.4%. The remaining survivors (23 patients) were followed-up the Surgical Outpatient Department for a period of 24 months. Follow-up of patients among survivors ranged from 3 to 24 months with a median of 8 months. At the end of the follow-up period, 14 (60.9%) patients were lost to follow-up and the remaining 9 (39.1%) patients died and their date of death was obtained from relatives. The patients survived for only a few weeks after clinical presentation with Sister Mary Joseph’s nodule. They survived for a median period of 28 weeks (range, 2 to 64 weeks). The nodule recurred in 6 (26.1%) patients after complete excision.

## Discussion

Sister Mary Joseph’s nodule refers to a palpable nodule bulging into the umbilicus as a result of metastasis of a malignant cancer in the pelvis or abdomen [1,5,6]. Sir Hamilton Bailey first described the term “Sister Mary Joseph’s nodule” in 1949 in honor of Sister Mary Joseph who was a nurse superintendent and a surgical assistant of Dr William Mayo at St Mary's Hospital (presently the Mayo Clinic) in Rochester Minnesota, USA from 1890 to 1915. Sister Mary Joseph was the first to note the link between umbilical nodules and intra-abdominal malignancy [2-4]. Since then, more than 400 cases of Sister Mary Joseph's nodule have been described in the literature [17]. It is estimated that 1 to 3% of patients with abdomino-pelvic malignancy could present with a Sister Mary Joseph’s nodule [5-7].

In this review, the median age of patients at presentation was 46 years, which is lower than the age reported in the literature [6,19]. A study performed in India by Al-Mashat and Sibiany [19] reported the mean age of patients at diagnosis to be 50.6 years (range, 18–87 years). Other studies reported older age at presentation [19,20]. We could not establish the reason for this age difference.

Epidemiological studies revealed that Sister Mary Joseph's nodule predominates in women [20,21]. This observation is at variant with our findings in the present study which showed male predominance. The reason for this gender difference remains unclear.

The gastrointestinal tract is the most common location of the primary neoplasm (35 to 65%), followed by a gynecological origin (12 to 35%) [1]. The common sites in decreasing order of frequency are: stomach (25%), colorectal (10%), and pancreas (7%) [1,19]. In females, ovarian cancer is the most common primary site, of which serous papillary cystadenocarcinoma is the most frequent (34%) [1,11]. As reported in the literature [11], the stomach was the most common primary site in this study. Primary tumors in many other sites have been reported to lead to Sister Mary Joseph's nodule, including gallbladder, liver, breast, lung, prostate, penis, peritoneum, lymphoma, bladder, kidney, endometrium, cervix, vagina, vulva, and fallopian tube [11,19]. In 15 to 30% of the cases, the source of the primary site of the tumor is unknown [8-10]. This finding concurs with our study in which the primary site was not known in 29.4% of cases.

Sister Mary Joseph's nodule can be the first manifestation of an underlying malignancy or an indication of a recurrence in a patient with a previous malignancy [22]. In the present study, 1 patient developed metastatic umbilical nodule 3 years after laparotomy for colonic cancer. Al-Mashatand Sibiany [19] reported 1 patient who developed Sister Mary Joseph's nodule 3 years after laparoscopic cholecystectomy. Direct implantation following laparoscopy has been reported in the literature to be another mode of spread of intra-abdominal tumors to the umbilicus [23].

The majority of patients in this study presented late with advanced stage of cancer which is in keeping with other studies performed in developing countries [19,22]. Late presentation in our study may be attributed to lack of awareness of the disease, a low standard of education, a lack of accessibility to healthcare facilities, and a lack of advanced diagnostic investigations such as MRI and PET and a panel of tumor markers in this region. Late presentation of cases is an area of cancer care in our center that requires urgent attention. Detecting primary cancer at an early stage contributes to improved chances for successful treatment and thus for survival.

The potential differential diagnosis to be considered when evaluating a patient with Sister Mary Joseph's nodule includes benign causes such as endometriosis, melanocytic nevi, fibroepithelialpapillomas, dermatofibroma, fibroma, epithelial inclusion cyst, urachal duct cyst, seborrheic keratosis, pilonidal sinus, keloid, foreign body, granuloma, myxoma, omphalitis, polyp, abscess, and umbilical hernia [5,11,24]. Additional differential diagnosis includes primary malignant umbilical tumors, which are exceedingly rare, accounting for 17% of the cases and including melanomas, basal cell carcinomas, squamous cell carcinomas, myosarcomas, and adenocarcinomas [5,11].

The spread of metastatic cancer to the umbilical region has been postulated to occur in several ways. These include direct extension from a contiguous tumor (the most important), hematogenous (arterial and venous), lymphatic and direct extension along the vestigial remnants of embryonal ligaments including the round ligament, the urachus, the vitellointestinal duct remnant, and the obliterated vitelline artery [24-26]. In addition, direct implantation following laparoscopy is another mode of spread of tumors to the umbilicus [23].

Patients with Sister Mary Joseph's nodule often present with a painful lump with irregular margins and hard consistency. The surface may be ulcerated and necrotic with either blood, serous, purulent, or mucous discharge from it. The size of the nodule usually ranges from 0.5 to 2 cm, although some nodules may reach up to 10 cm in size [1,6]. The median size of the nodule in our study was 3 cm and the vast majority of patients (82.4%) presented with large nodule >2 cm in size and more than 70% of nodules were ulcerated.

Various imaging modalities can aid in establishing the diagnosis, such as ultrasonography, CT, MRI, and PET. Once Sister Mary Joseph's nodule is discovered, a biopsy – either excisional or fine needle aspiration cytology – is mandatory to establish diagnosis and to find the possible primary site. The histopathological evaluation may show characteristics of the underlying tumor, or they may have a more anaplastic appearance. In the situation of an anaplastic tumor, immunohistochemical marker studies and ultrastructural examination may help delineate the tissue of origin [27]. In the present study, the diagnosis of primary cancer was performed by gastrointestinal endoscopies (that is,oesophagogastroduodenoscopy) and imaging modalities (that is, abdominal ultrasound, CT scan and barium studies) and histopathological examination of the biopsy specimen was performed to confirm the diagnosis. Advanced diagnostic investigation such as MRI, ERCP, PTC, PET and a panel of tumor markers was not performed in any of our patients as these facilities are not available at our center.

The most common histopathological type in our patients was adenocarcinoma, accounting for 88.2% of cases, which is consistent with that reported in the literature [11,19]. Other histopathological types include non-Hodgkin's lymphoma, squamous cell carcinoma, anaplastic carcinoma and cholangiocarcinomawhich is similar to the worldwide experience [11-14,19]. More than 50% of tumors in this study were poorly differentiated.

The presence of Sister Mary Joseph's nodules usually signifies an advanced, metastasizing cancer and, therefore, a poor prognosis. The finding of a metastatic nodule at the umbilical site almost certainly establishes the inoperability of the patient [28]. This conclusion was disputed by others who believe that the existence of Sister Mary Joseph's nodule is insufficient proof of widespread metastatic disease that would prevent an operation. Uncommonly, Sister Mary Joseph's nodule may represent a solitary metastasis or possibly a primary tumor that has not yet metastasized [29,30].

Sister Mary Joseph's nodule usually represents widespread metastasis and treatment is commonly palliative. Several authors have advocated wide excision with extensive search for the primary lesion [30,31], radiotherapy [32], and surgery with adjuvant therapy [26]. Majmudar and colleagues [26] have shown that patients treated aggressively with both surgery and adjuvant therapy live for an average of 17.6 months, which is more than with surgery alone (7.4 months), adjuvant therapy alone (10.3 months), or no treatment (2.3 months). An aggressive surgical approach combined with chemotherapy may improve survival [1]. In our series, surgical interventions were of a palliative nature, without curative intent, as all patients had either locally advanced or metastatic disease. Wide nodule excision and gastro-jejunostomy were the most frequent surgical procedure performed in this study. Adjuvant chemotherapy was given to only 14.7% of cases.

Sister Mary Joseph's nodule has traditionally been considered a sign of advanced primary malignancy with an associated poor prognosis; the average survival time has been reported to be 11 months with <15% of the patients surviving >2 years [19]. In some patients, however, depending on the state of the primary neoplasm and the patient's general condition, surgery and/or chemotherapy may improve survival [1]. In agreement with other studies [19,22], our patients survived for a median period of 28 weeks. This very short survival is in keeping with the advanced and metastatic nature of the primary cancer.

The potential limitation of this study is the fact that information about some patients was incomplete in view of the retrospective nature of the study and this might have introduced some bias in our findings.

## Conclusion

Sister Mary Joseph’s nodule of the umbilicus is not rare in our environment and often represents manifestation of a variety of advanced intra-abdominal malignancies. The majority of the patients present at a late stage with advanced disease. The patient's survival is very short leading to a poor outcome. We believe that if the primary cancer is detected at an early stage, the prognosis may improve. A careful examination of all umbilical lesions is recommended, especially in those patients with gastrointestinal and genitourinary tract malignancies. All umbilical mass lesions should be biopsied to determine the pathological nature of the lesion. An aggressive surgical approach combined with chemotherapy treatment may be considered to offer the patient the best survival probability.

## Abbreviations

CT: computed tomography; CUHAS: Catholic University of Health and Allied Sciences; MRI: magnetic resonance imaging; PET: positron emission tomography.

## Competing interests

The authors declare that they have no competing interests. The study had no external funding. Operational costs were met by the authors.

## Authors’ contributions

PLC conceived the study and participated in the literature search, writing the manuscript, editing and submission of the manuscript. JBM, PFR and MDM participated in study design, data analysis, manuscript writing and editing. All the authors read and approved the final manuscript.
